# The effects of CD148 Q276P/R326Q polymorphisms in A431D epidermoid cancer cell proliferation and epidermal growth factor receptor signaling

**DOI:** 10.1002/cnr2.1566

**Published:** 2021-11-17

**Authors:** Lilly He, Keiko Takahashi, Lejla Pasic, Chikage Narui, Philipp Ellinger, Manuel Grundmann, Takamune Takahashi

**Affiliations:** ^1^ Division of Nephrology and Hypertension Vanderbilt University Medical Center Nashville Tennessee USA; ^2^ Department of Biochemistry Vanderbilt University Nashville Tennessee USA; ^3^ Bayer AG Research & Development, Pharmaceuticals Wuppertal Germany

**Keywords:** A431D cells, CD148, cell proliferation, EGFR, epidermoid cancer, polymorphism

## Abstract

**Background:**

CD148 is a transmembrane protein tyrosine phosphatase that is expressed in multiple cell types. Previous studies have shown that CD148 dephosphorylates growth factor receptors and their signaling molecules, including EGFR and ERK1/2, and negatively regulates cancer cell growth. Furthermore, research of clinical patients has shown that highly linked CD148 gene polymorphisms, Gln276Pro (Q276P) and Arg326Gln (R326Q), are associated with an increased risk of several types of cancer. However, the biological effects of these missense mutations have not been studied.

**Aim:**

We aimed to determine the biological effects of CD148 Q276P/R326Q mutations in cancer cell proliferation and growth factor signaling, with emphasis on EGFR signaling.

**Methods:**

CD148 forms, wild‐type (WT) or Q276P/R326Q, were retrovirally introduced into A431D epidermoid carcinoma cells that lacks CD148 expression. The stable cells that express comparable levels of CD148 were sorted by flow cytometry. A431D cells infected with empty retrovirus was used as a control. CD148 localization, cell proliferation rate, EGFR signaling, and the response to thrombospondin‐1 (TSP1), a CD148 ligand, were assessed by immunostaining, cell proliferation assay, enzyme‐linked immunosorbent assay, and Western blotting.

**Results:**

Both CD148 forms (WT, Q276P/R326Q) were distributed to cell surface and all three cell lines expressed same level of EGFR. Compared to control cells, the A431D cells that express CD148 forms showed significantly lower cell proliferation rates. EGF‐induced EGFR and ERK1/2 phosphorylation as well as cell proliferation were also significantly reduced in these cells. Furthermore, TSP1 inhibited cell proliferation in CD148 (WT, Q276P/R326Q)‐expressing A431D cells, while it showed no effects in control cells. However, significant differences were not observed between CD148 WT and Q276P/R326Q cells.

**Conclusion:**

Our data demonstrates that Q276P/R326Q mutations do not have major effects on TSP1‐CD148 interaction as well as on CD148's cellular localization and activity to inhibit EGFR signaling and cell proliferation.

## 1 INTRODUCTION

CD148 (also known as DEP‐1, PTPη, or PTPRJ) is a receptor‐type protein tyrosine phosphatase (PTP) that is expressed in a variety of cell types, including epithelial cells, endothelial cells, and hematopoietic cells.^1^, ^2^, ^3^ A body of literature has demonstrated that CD148 has a tumor suppressing activity. First, CD148 is down‐regulated in cancer cells or neoplastic tissues, including thyroid, breast, renal, cervical, gastric, and pancreatic cancers,^4^, ^5^, ^6^, ^7^, ^8^, ^9^ while CD148 is upregulated on the differentiation of cancer cells^5^, ^10^ or with the cancer‐protective nutrients.^11^ Second, introducing CD148 into cancer cells strongly suppresses their cell proliferation and colony formation in culture and tumor growth in vivo, including breast, thyroid, pancreatic, renal, lung, gastric and colon cancer cells,^4^, ^5^, ^6^, ^7^, ^9^, ^10^, ^11^, ^12^ while CD148 knockdown promotes cancer cell proliferation and migration in culture and tumor growth in vivo.^7^, ^8^, ^9^, ^11^, ^13^ Last, CD148 expression level is inversely correlated with the survival rate in breast, renal, gastric, and lung cancers.^5^, ^7^, ^9^, ^12^

In support of CD148's tumor suppressing activity, a body of literature has shown that CD148 dephosphorylates growth factor receptors and their signaling molecules that play a major role in cancer cell growth, including epidermal growth factor receptor (EGFR),^9^, ^14^, ^15^, ^16^, ^17^ hepatocyte growth factor receptor (HGFR),^18^ platelet‐derived growth factor receptor‐β (PDGFRβ),^19^, ^20^ vascular endothelial growth factor receptor‐2 (VEGFR‐2),^15^, ^16^, ^21^ ERK1/2,^22^, ^23^ PLCγ1,^6^, ^24^ and p85 subunit of PI3‐kinase.^25^ It is of note that CD148 has been identified as a major EGFR‐targeting PTP.^14^, ^17^ Recently, soluble thrombospondin‐1 (TSP1) or syndecan‐2 (SDC2) was shown to serve as a ligand for CD148.^15^, ^26^

The genetic evidence also supports the role of CD148 as a tumor suppressor. PTPRJ (CD148) was identified as the unique candidate gene for the mouse colon‐cancer susceptibility locus *Scc1*.^27^ Somatic inactivating PTPRJ mutations were identified in 19% cases of canine malignant melanoma.^28^ In humans, loss of heterozygosity (LOH) of PTPRJ is frequently observed in colon, lung, breast, and thyroid cancers and Non‐Hodgkin's lymphoma.^27^, ^29^, ^30^, ^31^ Furthermore, several studies have implicated the PTPRJ polymorphisms in susceptibility to human cancers, which cause missense mutations in the extracellular domain of CD148. The PTPRJ genotype homozygous for Gln276Pro (Q276P) or Asp872Glu (D872E) allele are more frequently observed in thyroid carcinoma patients than in healthy individuals.^29^, ^32^ In a case–control study, one or more of the combination genotypes of Q276P and R326Q were associated with the increased risks for various types of cancers, including lung, head and neck, colorectal, and esophageal cancers.^33^ Recent pooled analysis also showed that R326Q genotype significantly increases the risk of human cancers.^34^ Interestingly, Q276P and R326Q were shown to be in perfect linkage disequilibrium.^29^, ^35^ On the other hand, several studies have shown that Q276P and/or R326Q genotype show no impacts on human cancer risks, including colon, breast, and thyroid cancers.^35^, ^36^, ^37^, ^38^, ^39^ Thus, conflicting results were also reported with these polymorphisms. It was recently shown that the haplotype impacts whether the polymorphism confers a cancer risk.^40^ Although these polymorphisms can be found in various populations, the haplogroup‐based analysis has not been conducted. The conflicting results may be due to the haplotype of subjects. To date, the biological effects of these highly‐linked missense mutations, Q276P and R326Q, have not been investigated.

In this study, we assessed the biological effects of Q276P/R326Q polymorphisms on CD148's activity to suppress cancer cell proliferation and EGFR signaling by stably introducing wild‐type (WT) or mutated (Q276P/R326Q) CD148 to A431D epidermoid cancer cells that lack endogenous CD148 expression. Furthermore, we also assessed its effects on CD148 interaction with an extracellular ligand, thrombospondin‐1 (TSP1). Our data demonstrates the first evidence that Q276P/R326Q missense mutations show no major effects on cellular localization and activity of CD148 to inhibit cancer cell proliferation and EGFR signaling as well as to interact with its ligand TSP1.

## 2 METHODS

### 2.1 Antibodies

Immunoblotting: Anti‐CD148 goat polyclonal (AF1934) was from R&D Systems (Minneapolis, MN). Anti‐HA rat monoclonal was from Roche (Indianapolis, IN). Anti‐β actin mouse monoclonal (C4) was from Santa Cruz Biotechnology (Dallas, TX). Anti‐pERK1/2 (T202/Y204) monoclonal antibody (clone D13.14.4E), anti‐total ERK1/2 monoclonal antibody (clone 137F5), anti p‐EGFR (Y1068) rabbit polyclonal antibody and anti‐EGFR (D38B1) XP monoclonal antibody were from Cell Signaling Technology (Danvers, MA). IRDye 680RD goat anti‐mouse IgG, IRDye 680RD goat anti‐rat IgG, IRDye 800CW donkey anti‐goat IgG, IRDye 680LT donkey anti‐rabbit IgG, and IRDye 800CW donkey anti‐rabbit IgG were from Li‐COR Biosciences (Lincoln, NE). Immunofluorescence: Anti‐CD148 mouse monoclonal (D‐4) was Santa Cruz Biotechnology. Anti‐HA epitope mouse monoclonal (clone 16B12) was from BioLegend (San Diego, CA). Alexa Fluor 546 goat anti‐mouse IgG (A11030) was from Invitrogen (Carlsbad, CA). Enzyme‐linked immunosorbent assay (ELISA): Anti‐total ERK (clone 137F5) rabbit monoclonal was used as a capture antibody. Biotinylated anti‐pERK1/2 antibody and Streptavidin/HRP detection reagents were provided with the “Phospho‐ERK1 (T202/Y204)/ERK2 (T185/Y187) DuoSet IC ELISA” kit from R&D Systems (Minneapolis, MN). Anti‐total ERK1/2 (Human/Mouse) mouse monoclonal and goat anti‐mouse IgG/HRP detection reagents were provided with the “PathScan Phospho‐p44/42 MAPK Sandwich ELISA Antibody Pair” from Cell Signaling Technology. Flow cytometry: Phycoerythrin (PE)‐conjugated anti‐human CD148 mouse monoclonal (clone 143‐41) was from R&D Systems. PE‐conjugated anti‐human EGFR and PE‐conjugated isotype control was from BD Biosciences (San Jose, CA).

### 2.2 Cells

A431D epidermoid carcinoma cells was provided by Dr. Albert Reynolds (Vanderbilt University).^41^ A431D cells is known to be characterized by the lack of cadherin expression as well as by the high level of EGFR expression.^42^, ^43^ These phenotypes were confirmed prior to the study. The cells were cultured in DMEM (GIBCO Life Technologies, Grand Island, NY) supplemented with 10% FBS (Sigma Aldrich, St. Louis, MO), 1% l‐glutamine, and 100 U/ml penicillin and 100 mg/ml streptomycin (GIBCO Life Technologies) as described previously.^15^ Phoenix (CRL‐3213) cells and HEK293 (CRL‐1573) cells were purchased from American Type Culture Collection (ATCC, Manassas, VA) and cultured in DMEM supplemented with 10% FBS, 2 mM l‐glutamine and 1% penicillin/streptomycin, according to the ATCC protocol. Cells were cultured in a humidified atmosphere of 5% CO_2_ at 37°C.

### 2.3 Site‐directed mutagenesis and retroviral vector construction

The c‐terminally hemagglutinin (HA) epitope‐tagged CD148 cDNA^1^ was subcloned into the XhoI/EcoRI sites in pBlueScript II SK (+) vector (Agilent Technologies, La Jolla, CA). The Q5 Site‐Directed Mutagenesis Kit (New England BioLabs, Ipswich, MA) was used to introduce the Q276P and R326Q mutations. The following PCR primers were designed to generate each mutation: Q276P (Forward: TAT CTT CTA CCA TCA AAT AAG ACA, Reverse: CGG GTT GAT GTT GTA TTG AAC CCC); R326Q (Forward: CAG CAG TCC CAA GAC ACG GAA GTC, Reverse: GCC GGA GGA TGG GTC CAC AGG TCC). The mutagenesis reactions were performed sequentially to create Q276P/R326Q mutations. The correct mutations were confirmed by DNA sequencing. There were no additional mutations in CD148 cDNA sequence. The mutated CD148 cDNA was then subcloned into the XhoI/EcoRI sites in the LZRS‐IRES‐Zeo retroviral vector^44^ that contains a zeocin resistant cassette. The LZRS‐IRES‐Zeo vector that expresses WT CD148 has been prepared previously.^15^

### 2.4 Retroviral production and stable cell preparation

Retrovirus was produced by transfecting the constructed retroviral vectors (CD148 WT, Q276P/R326Q) into Phoenix packaging cells as described previously.^15^ The empty vector was used to produce control virus. In brief, Phonix cell transfection was performed using the calcium phosphate‐DNA coprecipitation method in the presence of chloroquine. About 60 h post transfection, viral particles were collected, passed through a 0.45 μM filter, then concentrated using Retro‐X™ Concentrator (Clontech, Mountain View, CA). The concentrated retroviruses were added to A431D cells, and stable cells were selected with 400 μg/ml Zeocin (Invitrogen, Carlsbad, CA). The Zeocin‐resistant cells were stained with a PE‐conjugated anti‐CD148 antibody (R&D Systems), and the stable cells that express comparable levels of CD148 were sorted using a BD FACSAria II flow cytometer (BD Biosciences) as described.^15^ Control A431D cells were generated by the infection of retrovirus that was produced with the empty retroviral vector.

### 2.5 Flow cytometry

The cells were dissociated with 0.05%Trypsin, 0.53 mM EDTA (Corning, Manassas, VA), washed with PBS containing 0.5% Bovine Serum Albumin (BSA) (Sigma Aldrich), resuspended in cold 0.5% BSA‐PBS, and 2 × 10^5^ cells were incubated with 5 μl of PE‐conjugated anti‐human CD148 mouse monoclonal (clone 143‐41, R&D Systems) or 10 μl of PE‐conjugated anti‐human EGFR (BD Biosciences) for 45 min at 4°C. Cells were washed with 0.5% BSA‐PBS and analyzed using the three‐laser BD LSRII flow cytometer (Becton Dickinson, Franklin Lakes, NJ).

### 2.6 Immunoblotting

Cells were plated in 100 mm dishes and nearly confluent cells were lysed in RIPA buffer (50 mM Tris–HCl, pH 8.0, with 150 mM NaCl, 1.0% NP‐40, 0.5% sodium deoxycholate, 0.1% sodium dodecyl) (Sigma) containing a complete mixture of protease inhibitors (Roche).^16^ Clarified protein cell lysates were quantitated by BCA Protein Assay (Thermo Fisher Scientific). 4× Laemmli sample buffer (Bio‐Rad, Hercules, CA) was added to the lysates with 100 mM DTT, heated at 95°C for 10 min, and the equal amount of protein were separated by electrophoresis on a 7.5% Criterion™ TGX™ Precast Midi Gel (BioRad). The proteins were blotted on Trans‐Blot Turbo nitrocellulose membranes using Trans‐Blot TURBO Transfer System (Bio‐Rad) according to the manufacture's instruction. The membranes were blocked with Intercept Blocking Buffer (LI‐COR Biosciences) and incubated with anti‐CD148, anti‐HA, and anti‐β actin antibodies for 1 h at RT or overnight at 4°C. The membranes were washed and incubated with secondary antibodies for 1 h at RT. Fluorescence signals were detected and quantified using LI‐COR Odyssey CLx System (LI‐COR Biosciences) or Adobe Photoshop (San Jose, CA). For anti‐p‐ERK or p‐EGFR immunoblotting, cells were plated in 100 mm dishes at 40% confluency with growth medium, then serum was reduced to 0.5% FBS overnight. Cells were then treated with 10 ng/ml of EGF (PeproTech, Cranbury, NJ) for 5, 10, 15, and 30 min, immediately washed with cold PBS, and lysed in RIPA buffer (Sigma) containing protease and phosphatase inhibitor cocktails (Roche). Clarified protein cell lysates (25 μg for EGFR, 10 μg for pERK) were separated by electrophoresis on a 7.5% (EGFR) or 4%–15% (ERK1/2) Gradient Criterion™ TGX™ Precast Midi Gel (BioRad), and immunoblotting was carried out using anti‐pERK T202/Y204 or anti‐pEGFR Y1068 antibodies as described above. The antibodies were then stripped with 1.5× NewBlot Nitro Stripping Buffer (LI‐COR Biosciences) for 20 min at RT, blocked, and immunoblotting was carried out again using anti‐total ERK or anti‐total EGFR antibodies. The band intensity in Western blotting was quantified by LI‐COR Image Studio Software (LI‐COR Biosciences) and pEGFR/EGFR or pERK/ERK ratios were calculated.

### 2.7 Immunofluorescence

Cells were plated onto glass coverslips (Fisher Scientific, Hampton, NH) placed in a 12‐well plate, fixed in 2% paraformaldehyde (Santa Cruz Biotechnology) for 20 min at RT.^16^ For anti‐HA staining, cells were permeabilized with 0.2% saponin/PBS for 30 min at RT. Cells were incubated with the following primary antibodies for 1 h at RT: anti‐HA mouse monoclonal (1:500); anti‐CD148 mouse monoclonal (1:500). Then, cells were incubated with Alexa Fluor 546 goat anti‐mouse IgG (1:500) for 30 min at RT. Nuclear staining was carried out with 0.01 mM DRAQ5 (Thermo Fisher Scientific) according to the manufacture's protocol. Coverslips were mounted on glass slides with Fluoro‐Gel II (Electron Microscopy Sciences, Hatfield, PA). Confocal images were acquired using the Zeiss 510 confocal microscope (Carl Zeiss, Jena, Germany).

### 2.8 Enzyme‐linked immunosorbent assay

Cells were plated in 12‐well plates at 40% confluency with growth medium, and serum was reduced to 0.5% FBS or 0.1% FBS (serum starved condition) overnight. Then, 10 ng/ml of EGF was added to the medium for 5, 10, 15, and 30 min, the wells were emptied as quickly as possible, and lysed in ice‐cold RIPA buffer (40 μl/well, Sigma) containing protease and phosphatase inhibitor cocktails (Roche). An equal amount of clarified lysates was added to each ELISA well. A sandwich ELISA was used to assess ERK phosphorylation.^45^ A 384‐well plate was coated with anti‐total ERK and incubated overnight at 4°C. SmartBlock buffer (Candor Bioscience, Wangen, Germany) was then applied to the plate for 1 h before being discarded. An equal amount of cell lysates was added in quadruplicate and allowed to incubate for 1 h, washed with PBS + 0.1% Tween, and phosphorylated ERK was detected with biotinylated anti‐pERK1/2 antibody, followed by Streptavidin/HRP (DuoSet IC kit, R&D Systems). Simultaneously in separate wells, total ERK was detected with anti‐total ERK1/2 mouse monoclonal antibody and anti‐mouse IgG/HRP goat antibody (PathScan kit, Cell Signaling Technology). Amplex Red (Thermo Fisher Scientific) was added at a final concentration of 5 μg/ml with 0.007% H_2_O_2_, and fluorescence was measured via a plate reader (Ex: 560 nm, Em: 583 nm, Biotek Synergy Neo2, Winooski, VT). Based on fluorescence intensities, ratios of phospho ERK to total ERK were calculated to determine relative degrees of ERK activation within each lysate.

### 2.9 Cell proliferation assay

Cell proliferation assay was carried out as described previously.^15^ In brief, cells were plated in 96‐well plates (1.0–1.2 × 10^3^ cells per well) with growth medium. When the cells were attached (14–16 h after plating, day 0), serum was reduced to 0.1% FBS overnight (day 1). Then, cells were cultured in medium supplemented with 2.5% FBS (Figure 2), 0.3% FBS plus 60–240 ng/ml EGF (Figure 5), or 2.5% FBS plus 1–20 nM human TSP1 (Athens Research & Technology Inc., Athens, GA) (Figure 6). Cell number was assessed at the indicated time points using the Cy‐QUANT NF cell proliferation assay kit (Thermo Fisher Scientific). The medium was replaced every 2 days.

### 2.10 Statistical analysis

Data are expressed as means ± SEM. Statistical analysis was performed using Prism4 software (GraphPad Software Inc., La Jolla, CA). One‐way analysis of variance with post‐hoc Tukey honestly significant difference test was used to calculate the *p* value. *p* < .05 was considered as statistically significant.

## 3 RESULTS

### 3.1 Generation of A431D cells stably expressing CD148 (WT, Q276P/R326Q) forms

**FIGURE 1:**
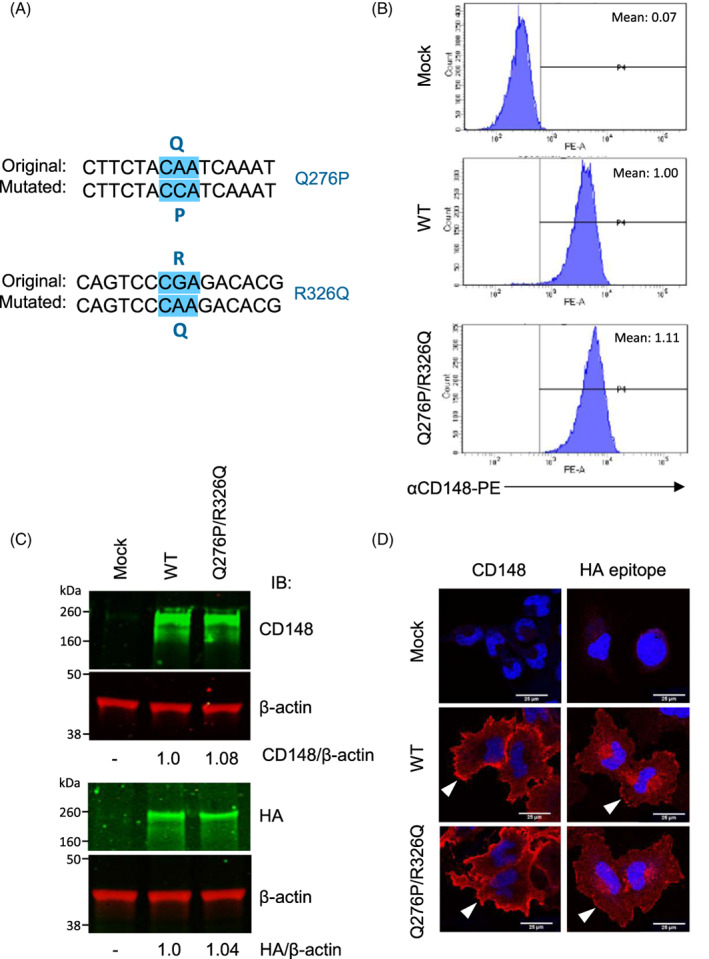
Generation of A431D cells that stably express CD148 forms (WT, Q276P/R326Q). (A) DNA sequence of mutated CD148 cDNA, confirming Q276P and R326Q mutations. (B) CD148 surface expression were examined in A431D‐CD148 (WT, Q276P/R326Q) cells and the control A431D cells that was generated by the infection of empty retrovirus (Mock) by flow cytometry using a PE‐conjugated anti‐CD148 antibody. Representative results of four independent experiments are shown. Mean fluorescence intensity is also shown. The fluorescence intensity of CD148 WT cells is expressed as 1.0. A431D‐CD148 WT and Q276P/R326Q cells express comparable levels of CD148. A431D‐Mock cells show no CD148 expression. (C) Expression of CD148 in A431D‐CD148 (WT, Q276P/R326Q) cells was also examined by Western blotting using anti‐CD148 or anti‐HA antibodies. Fifty micrograms of protein cell lysates were loaded into each well. The loading of proteins was assessed by anti‐β actin Western blot. The ratios of CD148 or HA to β actin are also shown. The ratios in A431D‐CD148 WT cells are expressed as 1.0. Representative results of four independent experiments are shown. (D) Immunolocalization of CD148 or HA epitope (both red) were examined in A431D‐Mock and A431D‐CD148 (WT, Q276P/R326Q) cells. The antibody against CD148 extracellular domain was used. Representative results of four independent experiments are shown. Scale bar = 25 μm. CD148 WT and Q276P/R326Q are similarly localized to the cell membrane (arrowheads). In anti‐HA immunostaining, there is perinuclear CD148 presence that is generally observed in stable cells

In order to assess the biological effects of Q276P/R326Q missense mutations, we generated the A431D cells that stably express same levels of CD148 forms (WT, Q276P/R326Q) using the retroviral vector and cell sorting. The A431D epidermoid carcinoma cells were used with the following reasons. First, A431D cells lack endogenous CD148 expression.^15^ Second, A431D cells highly express EGFR, a well‐studied substrate of CD148.^9^, ^14^, ^15^, ^16^, ^17^ Last, we have shown that CD148 inhibits cell proliferation and EGFR signaling in A431D cells.^15^, ^16^ The Q276P and R326Q mutations were introduced to the CD148 cDNA^15^ using site‐directed mutagenesis, which is c‐terminally epitope tagged with HA sequence, and successful mutations were confirmed by DNA sequencing (Figure 1A). Shown in Figure 1B, the generated A431D‐CD148 WT or Q276P/R326Q cells express same level of CD148 on cell surface, while control A431D cells that was generated with empty retroviral vector (hereinafter referred to A431D‐Mock cells) showed no CD148 expression. The expression level of CD148 in these stable cells were comparable to the CD148 level in cultured human primary endothelial cells^46^ (data not shown). The same level of CD148 expression in A431D‐CD148 WT and Q276P/R326Q cells was also shown by Western blotting (Figure 1C). Furthermore, the immunostaining of these cells with anti‐CD148 antibody (against ectodomain sequence) or anti‐HA antibody showed that both CD148 forms are similarly distributed to the cell membrane (Figure 1D). Since CD148 is c‐terminally epitope tagged with HA‐sequence, permeabilization of the cells was required for anti‐HA stain; therefore, immunoreactivity was also observed in cytoplasm. No immunoreactivities were observed in A431D‐Mock cells.

### 3.2 Cell proliferation rate in A431D‐CD148 (WT, Q276P/R326Q) cells

**FIGURE 2:**
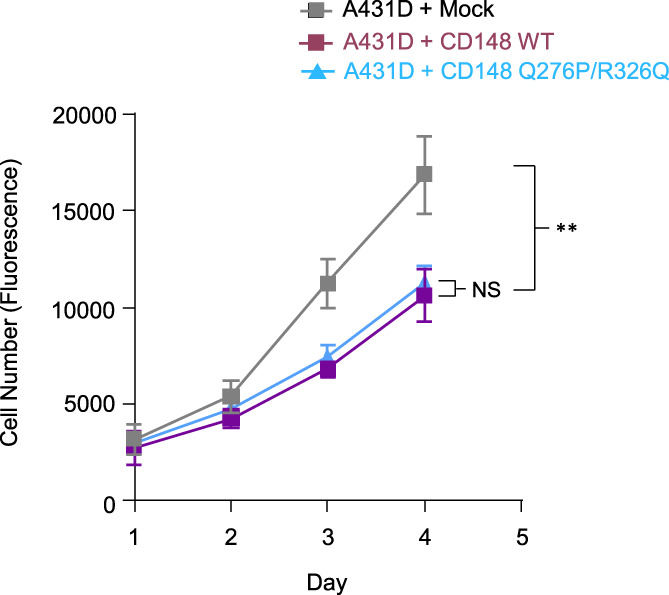
Cell proliferation rate in A431D‐CD148 (WT, Q276P/R326Q) cells. Cells (1.0 × 10^3^) were plated in 96‐well plates and serum starved (0.1% FBS) for overnight (day 1). Cells were then cultured in growth medium supplemented with 2.5% FBS. Cell number was assessed at day 1, 2, 3 and 4. Data are means ± SEM of quadruplicate determinations. Representative data of four independent experiments is shown. **p < .01. A431D‐CD148 (WT, Q276P/R326Q) cells showed lower cell proliferation rates than A431D‐Mock cells. No significant difference was observed between A431D‐CD148 WT and Q276P/R326Q cells

A body of literature demonstrated that CD148 expression inhibits cell proliferation in various type of cancer cells in culture.^4^, ^5^, ^6^, ^7^, ^9^, ^10^, ^11^, ^12^ In order to evaluate the biological effect of Q276P/R326Q polymorphisms, first we examined the cell proliferation rate of A431D‐CD148 WT or Q276P/R326Q cells as compared with A431D‐Mock cells. Shown in Figure 2, both A431D‐CD148 WT and Q276P/R326Q cells showed significantly lower cell proliferation rates than A431D‐Mock cells. However, there was no significant difference between the effects of CD148 WT and Q276P/R326Q on the extent to which they decrease the cell proliferation rate. The finding demonstrates that Q276P/R326Q polymorphisms do not alter the CD148 activity to inhibit A431D cancer cell proliferation.

### 3.3 EGFR signaling in A431D‐CD148 (WT, Q276P/R326Q) cells

**FIGURE 3:**
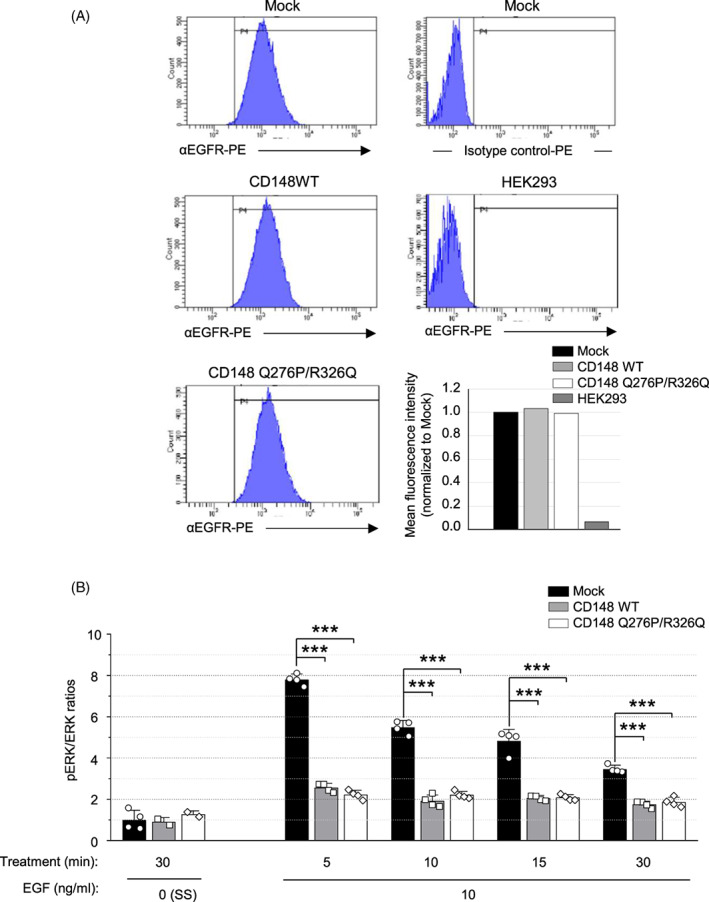
Expression of epidermal growth factor receptor (EGFR) and EGF‐induced ERK phosphorylation in A431D‐CD148 (WT, Q276P/R326Q) cells. (A) Cell surface expression of EGFR in A431D‐CD148 (WT and Q276P/R326Q) and A431D‐Mock (control) cells were examined by flow cytometry using a PE‐conjugated anti‐human EGFR antibody. HEK293 cells that do not express EGFR were used as a negative control. Mean fluorescence intensity is also shown (bottom right). The fluorescence intensity of A431D‐Mock cells is expressed as 1.0. Representative results of three independent experiments are shown. All three A431D cells showed comparable level of EGFR expression. (B) EGF‐induced ERK1/2 phosphorylation was examined in A431D‐CD148 (WT and Q276P/R326Q) and A431D‐Mock cells by ELISA as described in Section 2. Cells were treated with 10 ng/ml EGF for 5, 10, 15, and 30 min. The cells cultured in serum starved (SS) condition (0.1% FBS medium) for 30 min were used as a control. The p‐ERK/ERK ratios were normalized to serum‐starved (30 min) A431D‐Mock cells. Representative results of five independent experiments are shown. Data are means ± SEM of quadruplicate measurements. ***p < .001

**FIGURE 4:**
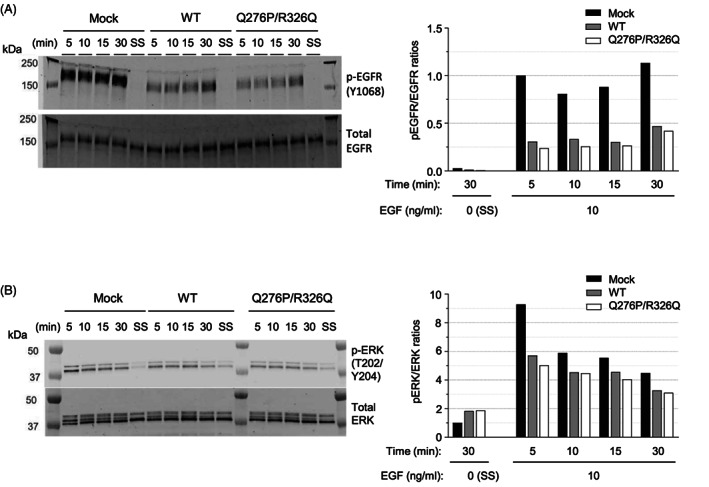
Immunoblotting to assess EGF‐induced epidermal growth factor receptor (EGFR) and ERK1/2 phosphorylation in A431D‐CD148 (WT, Q276P/R326Q) cells. (A) and (B) EGF‐induced EGFR and ERK1/2 phosphorylation was examined in A431D‐CD148 (WT, Q276P/R326Q) and A431D‐Mock cells by Western blotting as described in Section 2. Cells were plated in 100 mm dishes, serum was reduced (0.5% FBS), then cells were treated with 10 ng/ml EGF for 5, 10, 15, 30 min. The cells cultured in serum starved (SS) condition (0.1% FBS medium) for 30 min were used as a negative control. The p‐EGFR/EGFR ratios were normalized to A431D‐Mock cells treated with EGF for 5 min as EGFR phosphorylation was undetectable in serum starved (0.1% FBS) cells. The p‐ERK/ERK ratios were normalized to serum‐starved A431D‐Mock cells. Representative results of five independent experiments are shown

A body of literature has shown that CD148 dephosphorylates EGFR and suppress EGF signaling, especially ERK1/2 phosphorylation.^9^, ^14^, ^15^, ^16^, ^17^, ^22^ We therefore examined the effect of the Q276P/R326Q polymorphisms on CD148 activity to suppress EGFR signaling. First, we assessed the EGFR expression in A431D‐CD148 (WT, Q276P/R326Q) and A431D‐Mock cells by flow cytometry. Shown in Figure 3A, flow cytometric examination showed that cell surface expression of EGFR in A431D‐CD148 WT or Q276P/R326Q cells are comparable to that in A431D‐Mock cells, and there is no difference in surface EGFR levels between these two CD148 stable cells. Isotype control antibody and HEK293 cells that do not express EGFR^47^ were used as negative controls. These results were also confirmed by Western blotting (Figure 4A), which shows comparable levels of EGFR expression in A431D‐Mock, CD148WT, and Q276P/R326Q cells.

To investigate EGFR signaling in A431D‐CD148 WT or Q276P/R326Q cells, next we stimulated these cells along with A431D‐Mock cells with 10 ng/ml of EGF, and assessed the phosphorylation of ERK, a major downstream target in EGFR signaling,^48^ by ELISA. The dosage of EGF was determined by a preliminary dose–response experiment where EGF treatment induced ERK phosphorylation in all three cell lines in a dose‐dependent manner, which peaked at 5 min and decreased after 10 min of stimulation (Figure S1). Shown in Figure 3B, A431D‐CD148 cells, either WT or Q276P/R326Q, showed lowered EGF‐induced ERK phosphorylation compared to A431D‐mock cells. There was no significant difference in the activity between two CD148 forms, indicating that Q276P/R326Q does not alter the CD148 activity to suppress EGFR signaling. We also examined EGFR (Y1068) and ERK (T202/Y204) phosphorylation in these cells by Western blotting. The Y1068 is a key autophosphorylation tyrosine residue that reflects EGFR activation and serves as a Grb2 binding site inducing ERK1/2 activation.^49^, ^50^ Shown in Figure 4A,B, Western blotting data further confirmed the ELISA data of ERK phosphorylation and also showed that A431D‐Mock cells have more robust EGFR phosphorylation compared to A431D‐CD148 (WT, Q276P/R326Q) cells. However, evident difference was not observed in the levels of EGFR phosphorylation between A431D‐CD148 WT and Q276P/R326Q cells. CD148 expression exhibited a stronger effect on EGFR phosphorylation than ERK phosphorylation. This would be because CD148 more directly targets activated EGFR, while ERK phosphorylation is induced not only by EGF but also by other signaling events. Collectively, our data suggest Q276P/R326Q polymorphisms do not have major effects on CD148's activity to suppress EGFR signaling in A431D cells.

### 3.4 EGF‐induced cell proliferation in A431D‐CD148 (WT, Q276P/R326Q) cells

**FIGURE 5:**
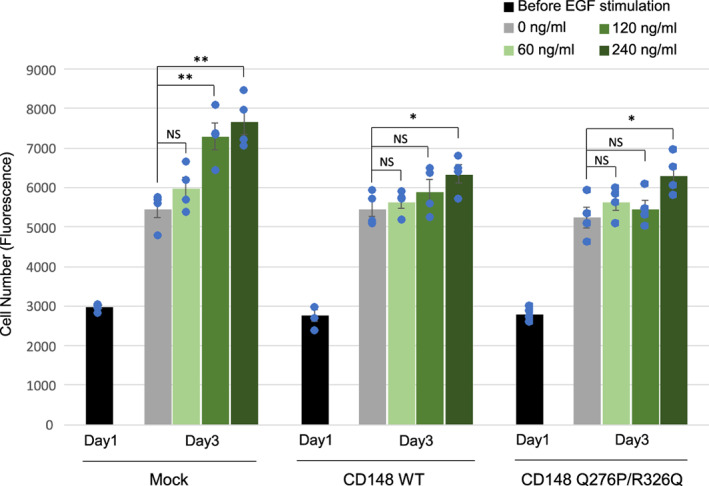
EGF‐induced cell proliferation in A431D‐CD148 (WT, Q276P/R326Q) cells. Cells (1.2 × 10^3^) were plated in 96‐well plates (day 0), then serum starved (0.1% FBS) for overnight (day 1). Cells were then treated with 60 ng/ml, 120 ng/ml, and 240 ng/ml EGF in growth medium supplemented with 0.3% FBS. Cell number was assessed at day 1 (before EGF stimulation) and day 3. Representative results of four independent experiments are shown. Data are means ± SEM of quadruplicate determinations. **p < .01, *p < .05

We further examined the effect of CD148 Q276P/R326Q polymorphisms on EGF‐induced cell proliferation in A431D cells. Shown in Figure 5, EGF treatment increased cell proliferation in all three cell lines in a dose‐dependent manner. The larger effects were observed in A431D‐Mock cells than in A431D‐CD148 WT or Q276P/R326Q cells, indicating that both forms reduce EGF‐induced cell proliferation in A431D cells. There was no significant difference in cell proliferation on EGF stimulation between A431D‐CD148 WT and Q276P/R326Q cells. The finding demonstrates that Q276P/R326Q polymorphisms do not alter CD148 activity to suppress EGF‐induced cell proliferation in A431D cells. It is of note that much higher dose of EGF was required to induce cell proliferation in A431D cells, while 10 ng/ml of EGF was sufficient to induce EGFR and ERK phosphorylation. EGFR is activated in A431D cells when they are cultured in growth medium, perhaps due to very high level of EGFR expression (ligand‐independent activation)^15^; therefore, the cells may be tolerant against EGF stimulation. Indeed, several reports have shown that high dose (10–20 nM; 60–120 ng/ml) EGF is required to induce cell proliferation in A431 cells.^51^, ^52^, ^53^

### 3.5 The responses of A431D‐CD148 (WT, Q276P/R326Q) cells to thrombospondin‐1

**FIGURE 6:**
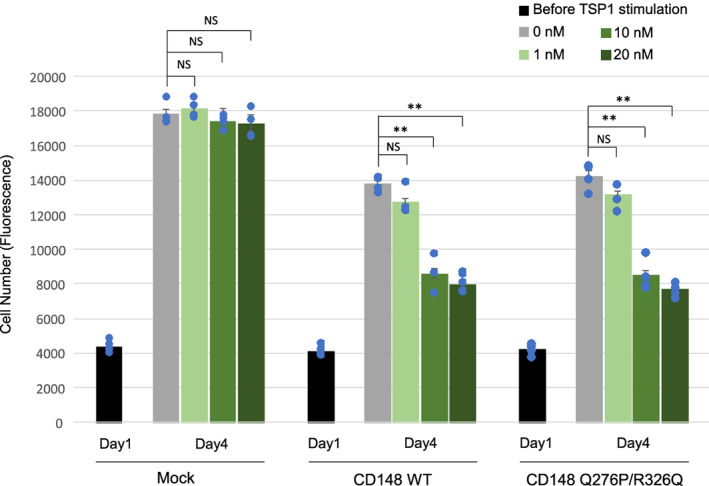
Effects of TSP1 on cell proliferation in A431D‐CD148 (WT, Q276P/R326Q) cells. Cells (1.0 × 10^3^) were plated in 96‐well plates (day 0), then serum starved (0.1% FBS) for overnight (day 1). Cells were then treated with 1, 10, 20 nM of TSP1 in growth medium supplemented with 2.5% FBS. Cell number was assessed at day 1 (before TSP1 stimulation) and day 4. Representative results of four independent experiments are shown. Data are means ± SEM of quadruplicate determinations. **p < .01

CD148 is composed of an extracellular segment of fibronectin type III (FN3) repeats, a transmembrane domain, and a single intracellular PTP domain.^54^ The Q276P/R326Q missense mutations are in the third FN3 repeat (UniProtKB‐Q12913); therefore, it may serve as an interface between the extracellular ligand and CD148. We have recently shown that soluble thrombospondin‐1 (TSP1) binds to the CD148 ectodomain, and its binding increases the CD148 catalytic activity and inhibits cell proliferation of A431D‐CD148 WT cells.^15^, ^16^ Therefore, next we asked if Q276P/R326Q mutations alter TSP1‐CD148 inhibition of A431D cell proliferation. Shown in Figure 6, TSP1 dose‐dependently inhibited cell proliferation in A431D‐CD148 WT cells, while it showed no effects in A431D‐Mock cells as described previously.^15^, ^16^ Interestingly, similar effects were observed in A431D‐CD148 Q276P/R326Q cells. There was no significant difference in TSP1 inhibition of cell proliferation between A431D‐CD148 WT and Q276P/R326Q cells, indicating that Q276P/R326Q mutations have no major effects on TSP1‐CD148 interaction.

Recent reports have shown that ectodomain of syndecan‐2 (SDC2) also binds to CD148 and triggers its catalytic activity.^55^, ^56^ According to the literature,^56^ we also examined the effects of Q276P/R326Q mutations on SDC2‐CD148 interaction using A431D cell proliferation assay. However, recombinant protein of SDC2 ectodomain (R&D Systems) showed no effects in all three cell lines (data not shown), even with 10.0 μg/ml dose, yet 0.5 μg/ml of SDC2 was shown to inhibit lung fibroblast activation and proliferation via CD148.^56^

## 4 DISCUSSION

The present study investigated the effects of CD148 Q276P/R326Q polymorphisms in cancer cell proliferation, growth factor signaling (with emphasis on EGFR signaling), and the CD148 ligand‐induced cancer cell growth inhibition by stably expressing same level of WT or Q276P/R326Q forms of CD148 in A431D epidermoid carcinoma cells that lack CD148 expression. Although Q276P and R326Q are predicted to cause torsional stress and loss of positive charge respectively,^27^ our data demonstrates that Q276P/R326Q mutations do not have major effects on CD148 interaction with its ligand TSP1 as well as on CD148's cellular localization and activity to inhibit cell proliferation and EGFR signaling in A431D cells.

Previous studies have shown that CD148 activity is regulated in two ways. Tarcic et al. have shown that CD148 interacts with EGFR when it is activated with its ligand EGF, dephosphorylates multiple tyrosine residues in activated EGFR, including Y1068, and suppresses the EGFR signaling (the ligand‐independent mechanism).^14^ On the other hand, we and others have demonstrated that CD148 catalytic activity is increased by the extracellular ligands such as TSP1 or SDC2.^15^, ^16^, ^26^, ^55^, ^56^ EGFR is highly expressed in A431D cells as well as in its parent A431 cells.^15^, ^16^, ^47^ In this cancer cells, EGFR is activated without EGF stimulation when they are cultured in growth medium, perhaps due to high level of EGFR expression, and we have shown that TSP1‐CD148 interaction suppresses this EGFR activity.^15^, ^16^ Furthermore, CD148 was shown to interact with‐ and dephosphorylate ERK1/2 that promotes cell proliferation.^22^ These would be a mechanism by which TSP1 inhibits cell proliferation in A431D‐CD148 WT or Q276P/R326Q cells. Although the CD148 regions that are responsible for CD148 interaction with EGFR or TSP1 are currently unknown, our data demonstrates that Q276P/R326Q polymorphisms do not alter the interactions of CD148 with activated EGFR or its ligand TSP1.

In this study, we normalized the expression levels of CD148 in the stable cells by cell sorting to assess the biological effects of Q276P/R326Q mutations. Therefore, the present data does not exclude the possibility that Q276P/R326Q mutations alter the expression level of CD148. However, this would be less likely. First, Q276P/R326Q mutations are in the extracellular domain, but not promoter region. Second, a recent study has shown that the surface expression level of CD148 is comparable in the platelets that carry WT or Q276P/R326Q alleles.^57^ In addition, we could not see obvious difference in CD148 levels (assessed by flow cytometry and Western blotting) between the A431D‐CD148 WT and Q276P/R326Q cells before sorting.

Given the facts that CD148 has a prominent activity in suppressing EGFR signaling^9^, ^14^, ^15^, ^16^, ^17^, ^22^ and that A431D cells express EGFR at high level, the present study has examined the effects of Q276P/R326Q mutations in EGFR signaling. However, CD148 was shown to dephosphorylate multiple growth factor receptors, including vascular endothelial growth factor receptor‐2 (VEGFR‐2),^15^, ^16^, ^21^ hepatocyte growth factor receptor (HGFR, c‐Met),^18^ platelet‐derived growth factor receptor‐β (PDGFRβ),^19^, ^20^ and GDNF receptor (RET).^58^ Furthermore, CD148 was shown to suppress cancel cell migration or angiogenesis.^13^, ^16^ It is possible that these mutations affect other cancer promoting events such as cancer cell migration, metastasis, and angiogenesis rather than cancer cell proliferation through targeting other molecules. In addition, we have shown that CD148 interacts with E‐cadherin and strengthens cell–cell adhesion by dephosphorylating p120 and β catenins.^46^ Last, there may be other extracellular ligands for CD148. In this context, it would be interesting to transplant these A431D cells into the immunodeficient mice and assess how Q276P/R326Q mutations alters A431D cancer cell growth in vivo, yet Q276 and R326 are not preserved in mouse CD148. Further studies would be required to investigate the effects of Q276P/R326Q mutations on these CD148 activities.

Given the fact that Q276P and R326Q polymorphisms are highly linked,^27^, ^29^, ^35^ the present study assessed the effects of Q276P/R326Q mutations. Mita et al. have shown that the subjects that have only one mutation, either Q276P or R326Q, exhibit high risks for certain cancers, yet these are rare types.^33^ Therefore, further investigation would also be required to assess the effects of each mutation. The frequency of Q276P or R326Q allele is ~17% (ALFRED database, Yale Center for Medical Informatics, New Haven, CT), and 36% of healthy subjects have Q276P/R326Q mutations and 3% of them has either Q276P or R326Q mutation.^33^ These mutations are more frequently observed in cancer patients. It would be important to determine its biological effects to better understand and treat the cancer patients.

## 5 CONCLUSION

This study has investigated for the first time the biological effects of CD148 Q276P/R326Q polymorphisms in cancer cell proliferation, growth factor (EGF) signaling, and the CD148 ligand‐induced cancer cell growth inhibition by introducing WT and mutated (Q276P/R326Q) CD148 into A431D epidermoid cancer cells. Our data demonstrates that Q276P/R326Q mutations show no major effects on functional interaction of CD148 with its ligand TSP1 as well as on CD148's cellular localization and activity to inhibit cell proliferation and EGFR signaling in A431D epidermoid cancer cells.

## CONFLICT OF INTEREST

Philipp Ellinger and Manuel Grundmann are full‐time employees at Bayer AG. Other authors have stated explicitly that there are no conflicts of interest in connection with this article.

## AUTHOR CONTRIBUTIONS

**Lilly He:** Formal analysis (equal); investigation (lead); methodology (equal); validation (equal); visualization (equal); writing – original draft (lead); writing – review and editing (equal). **Keiko Takahashi:** Formal analysis (equal); investigation (equal); methodology (equal); validation (equal); visualization (equal); writing – original draft (equal); writing – review and editing (equal). **Lejla Pasic:** Formal analysis (equal); investigation (equal); methodology (equal); validation (equal); visualization (equal); writing – original draft (equal); writing – review and editing (equal). **Chikage Narui:** Investigation (equal). **Philipp Ellinger:** Methodology (equal); writing – review and editing (equal). **Manuel Grundmann:** Methodology (equal); writing – review and editing (equal). **Takamune Takahashi:** Conceptualization (lead); formal analysis (equal); funding acquisition (lead); supervision (lead); validation (equal); visualization (equal); writing – original draft (equal); writing – review and editing (equal).

## ETHICS STATEMENT

This study was conducted with the institutional approval and guidelines of biosafety.

## Supporting information


**Figure S1** Dose‐dependent EGF‐induced ERK phosphorylation in A431D‐CD148 (WT, Q276P/R326Q) cells. A431D‐CD148 (WT, Q276P/R326Q) and A431D‐Mock cells were plated in 12‐well plates at 40% confluency with growth medium, then serum was reduced to 0.5% FBS for overnight. Cells were then treated with different doses (0, 0.1, 1.0, 10.0 ng/ml) of EGF for 5, 10, 15, and 30 min. Cell lysates were prepared, and ERK phosphorylation was assessed by ELISA as described in Section 2. The cells cultured in serum starved condition (0.1% FBS medium) were used as a control. The p‐ERK/ERK ratios were normalized to serum‐starved (5 min) A431D‐Mock cells. Representative results of three independent experiments are shown. Data are means ± SEM of duplicate measurements. Note: All three cell lines showed dose‐dependent ERK phosphorylation on EGF treatment. A431D‐Mock cells exhibited more robust ERK phosphorylation than A431D‐CD148 (WT, Q276P/R326Q) cells.

## Data Availability

The data that support the findings of this study are available from the corresponding author upon reasonable request.
