# Supplementary material for: The effects of CD148 Q276P/R326Q polymorphisms in A431D epidermoid cancer cell proliferation and epidermal growth factor receptor signaling
Source: Cancer Rep (Hoboken). 2021 Nov 17;5(9):e1566. doi: 10.1002/cnr2.1566 (PMC9458507; doi:10.1002/cnr2.1566)
Supplement: Supplementary file 1 — Figure S1 Dose‐dependent EGF‐induced ERK phosphorylation in A431D‐CD148 (WT, Q276P/R326Q) cells. A431D‐CD148 (WT, Q276P/R326Q) and A431D‐Mock cells were plated in 12‐well plates at 40% confluency with growth medium, then serum was reduced to 0.5% FBS for overnight. Cells were then treated with different doses (0, 0.1, 1.0, 10.0 ng/ml) of EGF for 5, 10, 15, and 30 min. Cell lysates were prepared, and ERK phosphorylation was assessed by ELISA as described in Section 2. The cells cultured in serum starved condition (0.1% FBS medium) were used as a control. The p‐ERK/ERK ratios were normalized to serum‐starved (5 min) A431D‐Mock cells. Representative results of three independent experiments are shown. Data are means ± SEM of duplicate measurements. Note: All three cell lines showed dose‐dependent ERK phosphorylation on EGF treatment. A431D‐Mock cells exhibited more robust ERK phosphorylation than A431D‐CD148 (WT, Q276P/R326Q) cells. [file CNR2-5-e1566-s001.pdf]

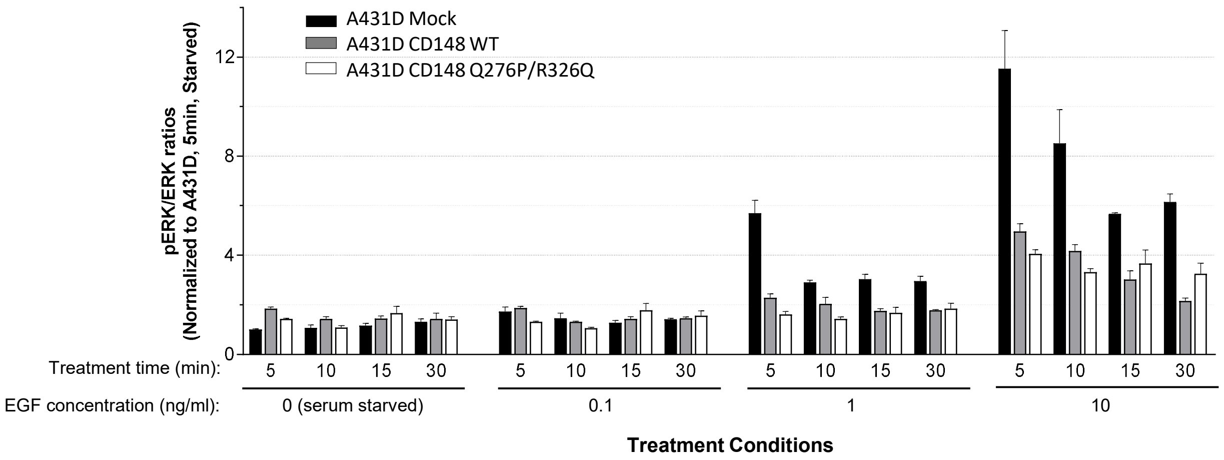

Supplemental Figure S1. Dose-dependent EGF-induced ERK phosphorylation in A431D-CD148 (WT, Q276P/R326Q) cells. A431D-CD148 (WT, Q276P/R326Q) and A431D-Mock cells were plated in 12-well plates at 40 % confluency with growth medium, then serum was reduced to 0.5% FBS for overnight. Cells were then treated with different doses (0, 0.1, 1.0, 10.0 ng/ml) of EGF for 5, 10, 15, and 30 min. Cell lysates were prepared, and ERK phosphorylation was assessed by ELISA as described in “Methods”. The cells cultured in serum starved condition (0.1% FBS medium) were used as a control. The p-ERK/ERK ratios were normalized to serum-starved (5 min) A431D-Mock cells. Representative results of three independent experiments are shown. Data are means ± SEM of duplicate measurements. Note: All three cell lines showed dose-dependent ERK phosphorylation on EGF treatment. A431D-Mock cells exhibited more robust ERK phosphorylation than A431D-CD148 (WT, Q276P/R326Q) cells.
